# Reclaiming ‘abnormal’ embryos after preimplantation genetic testing for aneuploidy: patients’ perspectives on transferring embryos against prior institutional advice

**DOI:** 10.1093/hropen/hoag025

**Published:** 2026-03-26

**Authors:** Shizuko Takahashi, Sonia Gayete-Lafuente, Elizabeth Choong, Lara Guijarro-Baude, David H Barad, Pasquale Patrizio, Michael Dunn, Julian Savulescu, Norbert Gleicher

**Affiliations:** Centre for Biomedical Ethics (CBmE), Yong Loo Lin School of Medicine, National University of Singapore, Singapore, Singapore; Department of Biomedical Ethics, Graduate School of Medicine, The University of Tokyo, Tokyo, Japan; Department of Biomedical Ethics & Humanities, University of Washington School of Medicine, Seattle, WA, USA; The Center for Human Reproduction, New York, NY, USA; The Center for Human Reproduction, New York, NY, USA; The Foundation for Reproductive Medicine, New York, NY, USA; Stem Cell Biology and Molecular Embryology Laboratory, The Rockefeller University, New York, NY, USA; The Center for Human Reproduction, New York, NY, USA; The Center for Human Reproduction, New York, NY, USA; The Center for Human Reproduction, New York, NY, USA; The Foundation for Reproductive Medicine, New York, NY, USA; The Center for Human Reproduction, New York, NY, USA; Department of Obstetrics and Gynecology, Gynecology and Reproductive Sciences, University of Miami, Miller School of Medicine, Miami, FL, USA; Centre for Biomedical Ethics (CBmE), Yong Loo Lin School of Medicine, National University of Singapore, Singapore, Singapore; Centre for Biomedical Ethics (CBmE), Yong Loo Lin School of Medicine, National University of Singapore, Singapore, Singapore; Uehiro Oxford Institute, University of Oxford, Oxford, UK; The Center for Human Reproduction, New York, NY, USA; The Foundation for Reproductive Medicine, New York, NY, USA; Stem Cell Biology and Molecular Embryology Laboratory, The Rockefeller University, New York, NY, USA; Department of Obstetrics and Gynecology, Medical University of Vienna, Vienna, Austria

**Keywords:** preimplantation genetic testing for aneuploidy (PGT-A), IVF, embryo mosaicism, aneuploid embryo transfer, reproductive ethics, procreative beneficence, qualitative research

## Abstract

**STUDY QUESTION:**

How do patients make sense of and justify the transfer of embryos labelled ‘abnormal’ by preimplantation genetic testing for aneuploidy (PGT-A)?

**SUMMARY ANSWER:**

Patients described a process of unlearning the authority of genetic testing and reframing their decisions as morally reasoned acts of reproductive agency—a reclaiming of possibility amid biomedical exclusion.

**WHAT IS KNOWN ALREADY:**

PGT-A is widely used in IVF, although its clinical utility and interpretation remain debated, and its claimed benefit on cumulative live birth rates unproven. Many clinics refuse transfer of embryos labelled ‘abnormal’, despite reports of healthy live births following transfer of selected embryos labelled ‘mosaic’ or ‘aneuploid’.

**STUDY DESIGN, SIZE, DURATION:**

Qualitative retrospective study of 21 participants (15 women and 6 men) conducted between 2019 and 2025 at a private fertility clinic associated with a research university in New York City, NY, USA.

**PARTICIPANTS/MATERIALS, SETTING, METHODS:**

Participants had previously been denied transfer of embryos labelled ‘abnormal’ by PGT-A at multiple clinics and sought care at the study site. Semi-structured interviews were conducted, audio-recorded, transcribed verbatim, and analysed using inductive, reflexive thematic analyses.

**MAIN RESULTS AND THE ROLE OF CHANCE:**

Among 21 participants, transfer outcomes included 6 healthy births following transfers of embryos labelled ‘abnormal’, 1 ongoing, 4 first-trimester miscarriages, 1 chemical pregnancy, and 12 failed implantations. One overarching theme ‘reclaiming possibility’, spanning four interrelated phases, was identified: (i) reassessing scientific authority of PGT-A; (ii) navigating institutional and relational constraints; (iii) embracing moral responsibility to give, or withhold, embryos a chance; and (iv) retrospective/anticipatory validation through a no-regrets stance. Participants resisted exclusionary classifications, pursued transfer despite refusals, and made sense of known, unknown, or adverse outcomes without equating results with the test’s predictive accuracy.

**LIMITATIONS, REASONS FOR CAUTION:**

Participants were self-selected from a single clinic that was willing to consider such embryo transfers; therefore, the findings may have limited transferability to other settings or to patients who accepted an original institutional transfer refusal.

**WIDER IMPLICATIONS OF THE FINDINGS:**

Patients’ narratives challenge assumptions that PGT-A results are determinative of embryo viability and highlight the ethical complexity of categorical transfer refusals by clinics. These findings support the need for transparent pre-test counselling, explicit institutional policies on embryo transfer, and patient-led pathways under robust informed consent. Integrating uncertainty-aware counselling and clear prenatal diagnostic planning may better align care with patients’ values and lived realities.

**STUDY FUNDING/COMPETING INTEREST(S):**

This work was supported by the Wellcome Trust [grant number: 226801] for Discovery Research Platform for Transformative Inclusivity in Ethics and Humanities Research (ANTITHESES). D.H.B. and N.G. are co-owners of several already awarded and still pending US patents, some claiming benefits from androgens, including DHEA, supplementation in women with low functional ovarian reserve, other infertility conditions, and perimenopausal hypo-androgenism-induced sexual dysfunction. Other patents relate to diagnostic and potential therapeutic benefits of AMH. N.G. is also a shareholder in Fertility Nutraceuticals LLC and N.G. receives patent royalties from Fertility Nutraceuticals, LLC. All other authors report no potential competing interests.

**TRIAL REGISTRATION NUMBER:**

N/A.

WHAT DOES THIS MEAN FOR PATIENTS? This study explains why some IVF patients choose to transfer embryos labelled ‘abnormal’ by preimplantation genetic testing for aneuploidy (PGT-A), that can include aneuploid and mosaic results with notable false-positive rates, and what helps them make this decision. We interviewed 21 patients who had been refused embryo transfer elsewhere and sought care at our clinic. Many had experienced repeated IVF failures, often after transferring embryos labelled ‘normal’, and felt they had few remaining options.

Participants weighed risks against their chance of having a child, questioned how much authority to give a screening label, and chose transfer with a clear understanding of uncertainty. They described unmet needs, including plain-language explanations of what PGT-A can and cannot show, clear clinic policies, and guidance about what happens if pregnancy occurs (such as further testing and support).

To make care truly patient-centred, clinics should provide honest pre-test counselling, transparent transfer policies, and clear pathways for decision-making under informed consent. Early, clear information can help patients make choices that align with their values and reduce distress.

## Introduction

Preimplantation genetic testing for aneuploidy (PGT-A) was initially introduced with the promise of improving IVF success rates by selecting ‘chromosomally normal’ embryos for transfer. Marketed globally as a tool to increase pregnancy and live birth rates, reduce miscarriages, and ensure the birth of ‘healthy’ babies, it has been particularly appealing to patients enduring the physical, emotional, and financial burdens of infertility. Over time, PGT-A, therefore, has become an often-routine add-on to IVF. Although PGT-A may shorten time to pregnancy in selected good-prognosis cohorts with larger embryo numbers, evidence of benefit in poorer-prognosis groups and effects on cumulative live birth remain contested (Cornelisse *et al.*, 2020; Yan *et al.*, 2021).

PGT-A testing involves performing a trophectoderm biopsy, typically 5–10 cells, from a blastocyst of approximately 250 cells, with the chromosomal profile of this small sample extrapolated to represent the entire embryo. Such an extrapolation has, however, significant limitations. Blastocyst-stage embryo aneuploidy is common and known to increase with maternal age, rising from 41.4% in women aged 25–34 years to 88.3% in women aged 41–46 years (Hochberg *et al.*, 2025). Moreover, mosaicism of embryos, which is defined as presence of more than one cell lineage—usually euploid and aneuploid—has greatly varied from ∼15–22% (Ai *et al.*, 2022).

Consequently, ‘abnormal’ reports may include mosaic or segmental findings of uncertain clinical significance, and biopsy results may not always mirror the chromosomal composition of the inner cell mass (Popovic *et al.*, 2018; Victor *et al.*, 2019; Cheng *et al.*, 2025), destined to become the future foetus, often leading to false-positive results of PGT-A which for patients can have potentially very negative consequences if IVF clinics either discard or just refuse transfer of such embryos with still often very good chances for a chromosomally normal pregnancy. At the blastocyst stage, concordance between trophectoderm biopsy and the remainder of the embryo is generally high for clearly euploid or aneuploid results, but lower for intermediate/mosaic calls, reflecting both biological mosaicism and sampling limits (Victor *et al.*, 2019; ESHRE Working Group on Chromosomal Mosaicism *et al.*, 2022; Kim *et al.*, 2022; ASRM, 2024). Evidence furthermore suggests that, downstream from blastocyst stage, human embryos—in contrast to the trophectoderm—often self-correct within the inner cell mass (Yang *et al.*, 2021).

Accordingly, a substantial proportion of embryos labelled as abnormal by PGT-A may in fact be capable of producing healthy pregnancies. Our centre was the first to report, in 2015, four chromosomally-normal pregnancies following the transfer of such by PGT-A as abnormal reported embryos (Gleicher *et al.*, 2015). Shortly thereafter, six additional cases were reported (Greco *et al.*, 2015).

Since then, further reports, including a cohort of 69 patients of very advanced median age and denied transfers elsewhere—were, as reported in 2022, transferred at our centre achieving 8 live births (11.6% per cycle start), thereby confirming beyond reasonable doubt that some embryos, by PGT-A diagnosed as abnormal-mosaic as well as abnormal-aneuploid, can result in chromosomally normal and overall healthy offspring (Barad *et al.*, 2022). These observations suggest that a subset of ‘abnormal’ calls may represent sampling variance or non-representative mosaicism and, therefore, such embryos should not be categorically excluded from transfer under robust counselling and consent (Orvieto *et al.*, 2020; Gleicher *et al.*, 2025a).

Many IVF clinics, however, still routinize PGT-A in IVF and may treat results as determinative, sometimes using testing as a *de facto* precondition for transfer and denying transfers of embryos reported as mosaic or aneuploid. These practices can steer patients prematurely toward repeat cycles or even toward third-party egg donation, at times without full disclosure of limitations and clinical management policies regarding PGT-A. What began as a tool for precision IVF has, therefore, for many patients become a mechanism of exclusion when additional cycles are not feasible or acceptable.

While acceptance of mosaic embryo transfer has significantly grown (Greco *et al.*, 2015; Peikoff, 2016; Zhang *et al.*, 2020; Molin *et al.*, 2025), such transfers remain nationally banned in several countries. Moreover, many IVF clinics, even if legally allowed, still choose not to transfer mosaic embryos (McGowan *et al.*, 2020; Chan *et al.*, 2022). Contemporary professional guidance acknowledges this heterogeneity and recommends structured counselling, triage, and prenatal diagnostic planning for selected mosaic transfers, while transfers reported as fully aneuploid remain rare (Chan *et al.*, 2022; ESHRE Working Group on Chromosomal Mosaicism *et al.*, 2022; ASRM, 2024; HFEA, n.d.). To avoid over-generalization, we refer to published surveys/guidance rather than centre-level assertions.

As patients unfortunately remain largely excluded from the debate, critics further argue that PGT-A lowers cumulative live birth rates across most age groups while benefiting only relatively small select subpopulations (Kucherov *et al.*, 2023) and that pursuit of ‘better outcomes’ can obscure ethically significant trade-offs. As Scriven has noted within such a context, a test that eliminates all embryos in an IVF cycle would, of course, prevent miscarriage simply by preventing pregnancy (Scriven, 2022).

For several reasons, these issues have recently drawn substantial public and legal scrutiny. Beyond legacy-media coverage by, for example *Time* magazine, (Ducharme, 2025) a series of class-action lawsuits filed in the USA since late 2024 allege that major PGT-A laboratories, and at least one clinic, misrepresented the PGT-A test’s accuracy (claimed to be 96–99%) and its clinical benefits,—including higher live birth rates, lower miscarriage rates, and faster time to pregnancy—while downplaying interpretive limits, and, therefore, leading to economic loss of many IVF patients and, in some cases in the disposal of potentially viable embryos. These developments have intensified calls for transparent, pre-test counselling, and clearer reporting standards.

From a patient’s perspective, decisions about embryos labelled ‘abnormal’ carry significant emotional and ethical weight. Embryos are often perceived as ‘virtual children’ (Lyerly *et al.*, 2006), embodying both hope and treatment burdens (De Lacey, 2005; Takahashi *et al.*, 2012). Therefore, decisions to discard embryos, even when embryos are labelled ‘abnormal’, are deeply emotional and ethically complex. A recent study by Prytkova *et al.* (2023) found that—unrelated to demographics or insurance status—nearly 40% of patients revised their embryo disposition directives between IVF cycles (Prytkova *et al.*, 2023), reflecting not indecision but the weight of choices about embryos that otherwise had the potential of becoming children. Together, these observations underscore the need for individualized counselling and robust, uncertainty-aware informed consents.

Patients who pursue embryo transfers are by no means indifferent to responsibility; their choices, indeed, reflect complex moral reasoning often overlooked in ethical critiques. While decisional needs in cases with mosaic embryos have been studied before (Cheng *et al.*, 2022), little is known about those who proceed with ‘abnormal’ embryos after refusals of transfer at their usual fertility clinics where they failed to conceive. By ‘ethical reasoning’, we refer to the process by which participants think through what is right, fair, or morally appropriate in their circumstances—analysing the dilemma, considering likely consequences, weighing relevant values (e.g. honesty, fairness, respect for autonomy, and harm/benefit, including responsibilities to prospective children and acceptable risk)—and arriving at a decision about whether and under what conditions transfer is permissible. The aim of this study is to examine how patients who transferred embryos labelled ‘abnormal’ by PGT-A interpret test results, navigate institutional barriers, and reason about risk and responsibility and to translate these insights into practical implications for pre-test counselling, result management, and policy.

We here present our findings and derive from them clinical and bioethical implications for uncertainty-aware counselling, result management, and policy.

## Methods

### Setting

This qualitative study was conducted at the Center for Human Reproduction (CHR), a private fertility clinic in New York City associated with a research university, between October 2019 and June 2025. The clinic considers transfers of embryos reported ‘abnormal’ by PGT-A under rigorous counselling and consent; for this study, ‘abnormal’ refers to laboratory reports of aneuploid, segmental aneuploid, or mosaic calls at the blastocyst stage.

### Ethics approval

Ethical approval was obtained from CHR Institutional Review Board (IRB No. ER 08012019; No. ER08292024).

### Participants and recruitment

All patients who decided to have their embryos labelled as abnormal transferred at our centre were eligible for participation and received an email invitation to undergo anonymous volunteer interviews regarding their clinical decision-making process. Eligible individuals were identified from a clinic registry of patients whose embryos had been reported ‘abnormal’ by PGT-A and subsequently transferred at CHR after transfer was declined at the originating clinic, except for one couple (Cases 8–9) who had shipped embryos but had not yet undergone transfer due to COVID-19 restrictions. A total of 21 individuals (15 women and 6 men) were willing to participate and recruited for the study. No restrictions to recruitment were placed on gender identity, race, sexual orientation, or outcome of the transfer. Recruitment occurred in two waves (2019–2020; 2024–2025) with a pandemic-related pause in between; IRB approval was renewed prior to resumption.

### Data collection

Semi-structured interviews were conducted from October 2019 to July 2025 using a guide based on previous research (Takahashi *et al.*, 2012) (Supplementary File S1). Participants were first asked an open-ended question about their IVF journey leading to transfer of PGT-A labelled abnormal embryos. All interviews occurred retrospectively, after embryos had been shipped to CHR; at interview, all participants knew their transfer outcome except one couple (Cases 8–9, awaiting transfer) and one participant (Case 19, 6-day post-transfer). Interviews were audiotaped and transcribed verbatim.

All interviews were conducted by the first author (S.T.), an Obstetrician–Gynecologist and Clinical Geneticist unaffiliated with the participants’ clinical care. Twenty-one interviews took place by videoconference, one by phone, and two in person. Informed consent was obtained, either written and/or oral and interviews averaged 48 min (range 20–81). Both partners were invited to participate, with couples choosing joint or separate sessions. One couple (Cases 14 and 15), were interviewed separately to encourage disclosure (Taylor and de Vocht, 2011).

### Analysis

All transcripts were analysed using ATLAS.ti (Version 25.0.1, last accessed 15 September 2025, Scientific Software Development GmbH, Berlin). We employed an inductive, reflexive thematic analysis (TA) (Braun and Clarke, 2006, 2022), chosen for its flexibility and its suitability for capturing the complexity of participants’ lived experiences. This approach supported interpretive depth and theoretical flexibility, particularly in exploring how participants made sense of uncertainty surrounding PGT-A ‘abnormal’ results and redefined reproductive agency. The study adopted a critical realist epistemology, assuming that participants’ accounts reflect both material realities and socially mediated meanings (Fletcher, 2017). This was particularly appropriate given the absence of a pre-existing conceptual framework to comprehend patients’ decisions to transfer embryos labelled as abnormal.

Initial interviews (2019–2020) were inductively coded and thematically refined through team review, peer debriefing, and interdisciplinary feedback. COVID-19 paused recruitment, and, upon resumption, the study scope expanded from reportedly mosaic to total aneuploid embryos, with IRB approval. Analysis found no substantive differences between groups, enabling a combined dataset. Initial coding was conducted by the first author (S.T.), with subsequent review by co-authors (S.G-L., E.C., L.G-B., D.B., N.G., M.D., and J.S.).

To enhance analytic quality, we adopted several strategies aligned with reflexive TA (Braun and Clarke, 2006, 2022). We practiced ongoing reflexivity through regular team discussions about assumptions and positionality, including how clinical and non-clinical perspectives on ‘abnormal’ embryo transfer might shape coding and theme development. To support deep familiarization and accurate representation of participants’ accounts, the lead researcher repeatedly returned to the audio recordings alongside transcripts. We also strengthened interpretive diversity through peer debriefing: 11 of the 15 anonymized transcripts and developing codes/themes were reviewed by non-medical researchers at our institutions (University of Tokyo and National University of Singapore) and preliminary themes were presented at international conferences and workshops to solicit critical feedback and refine the analytic story. Given the pandemic-related intermission, opportunities to organize additional full-transcript review by non-clinical colleagues were limited. Through latent coding, we attended not only to participants’ explicit statements but also to the implicit meanings, values, and assumptions underlying their accounts, interpreting what was said in light of what was implied through reasoning patterns, emotional tone, and contextual references. Discrepancies in code application were resolved through discussion and returning to the raw data, with a third researcher consulted when consensus could not be reached (J.S. and N.G.). A coding framework was iteratively refined through this process to ensure consistency.

Themes were actively constructed—not discovered—through recursive engagement with the data, attending to both semantic and latent dimensions (Braun and Clarke, 2006, 2022). While ATLAS.ti was used to facilitate transcript coding and data organization, theme development was conducted manually by printing coded segments and clustering them into conceptual categories. Data collection continued until we judged the dataset was sufficiently rich to address the research aims and support coherent, well-evidenced themes (Guest *et al.*, 2006). Given the retrospective design and the option of joint interviews, potential recall and response biases are acknowledged in the Limitations; we attempted mitigation via timeline-anchored prompts and comparison of theme expression across interview formats.

## Results

Demographics, reproductive history, ‘abnormal’ embryo transfer details and outcomes of participants are summarized in Table 1. The mean age of participants was 42 years. Most held a university degree, with four having a background in science or medicine. A majority had at least one child. Participants’ reasons for undergoing PGT-A included sex selection (n = 7), previous experience with trisomy (n = 3), and strong institutional recommendation at the IVF clinic (n = 11). The transfer outcomes for the 21 participants were: six live births following the transfer of an embryo labelled ‘abnormal’ (including one who had a combined transfer of an ‘abnormal’ and an untested embryo), four first-trimester miscarriages, one chemical pregnancy, and 12 failed implantations. When ongoing pregnancies were achieved after transfer of embryos labelled ‘abnormal’, newborns were clinically healthy at birth; when undertaken, invasive prenatal diagnosis did not confirm the reported abnormality.

**Table 1.** Demographic characteristics of the participants.

Cases	Age* at interview (years)	Gravida Para	Infertility diagnosis	Years of treatment	Reason for PGT-A	Age* at biopsy	No. IVF cycles	No. FET	PGT-A result	Karyotype at outcome	IVF outcome
Case 1	38	G1P1	n/a	1	Sex selection	36	2	FET1	47, XX, +14[mos]; 47, XY, +22[mos]	N/A	SAB, IF
Case 2	41	G1P2	n/a	4	Previous 21 T	38	6	FET1	44, XX, −15, −22; 45, XX, −22; 45, XX, −15	N/A	IF
Case 3	43	G1P0	n/a	1	Routine	39	4	FET1	47, XX, +19, dup(9)(p12p24); 45, XX, −15	N/A	IF
Case 4	44	G3P1	n/a	6	Routine	38	2	FET1	47, XX, +12; 45 XX, −22	47, XX, +12	SAB, IF
Case 5	39	G2P 2	PCOS	1	Sex selection	35	2	FET1	45, XY, −6[mos]	46, XY (*normal karyotype*)	2LB
								FET2	45, XY, −17[mos]	N/A	IF
Case 6	46	G7P2	n/a	4	Routine	40	5	FET1	45, XX, −21[mos]	N/A	Chemical pregnancy
Case 7	42	G2P1	n/a	5	Routine	41	2	FET1	46, XY, del (5) (pter-q22.3) [mos], dup (20) (pter-p11.23) [mos];	N/A	LB, IF
								FET2	44 XX, −6, −7; 46, XX, +9, −22 [mos] 46 XY, −15, +20; 48, XX, +15, +19 [mos]	N/A	IF
Case 8 and 9	45	G2P1 (1 with T18)	n/a	3	Previous T18	42	Contact lost	Anticipating FET	N/A	N/A	N/A
Case 10 and 11	38	G4P2	n/a	1	Sex Selection	33	1	FET1	46, XX, dup 11p15.5q22.2(102 Mb), del 11q22.2q25(32 Mb)	N/A	IF
Case 12 and 13	44	G2P2	n/a	6	Routine	41	4	FET1	48, XX, +11, +20, +dup(1)(q?)	46, XX (*normal karyotype*)	LB
Case 14 and 15	47	G4P3	n/a	6	Routine	43	4	FET1	46, XX, +14, −18	46, XX (*normal karyotype*)	LB
Case 16 and 17	34	G4P1	n/a	7	Routine	28	5	FET1	45, XY, −16;	N/A	IF
								FET2	45, XX, −11;	N/A	IF
								FET3	46, XY, −10p;	N/A	IF
								FET4	46, XX, −11q	N/A	IF
Case 18	39	G4P2	n/a	7	Routine	34	7	FET1	46, XX , 7q35-qter	46, XY (*normal karyotype*)	LB
Case 19	47	G1P0	PCOS	10	AMA	46	5	FET1	45, XY, −21	45, XY, −21	SAB (after interview)
Case 20 and 21	44	G0	n/a	5	Routine	43	30	FET1	47, XY, +19; 47, XY, +12; 44, XY, −8, −16	N/A	SAB

AMA, advanced maternal age; FET, frozen embryo transfer (the number of transfers performed at our clinic, 1st, 2nd, 3rd, and 4th); IF, implantation failure; LB, live birth; NIPT, non-invasive prenatal testing; SAB, spontaneous abortion; GxPx, total number of confirmed pregnancies + number of pregnancies carried to viable gestational age; mos, mosaicism; dup, duplication.

* Maternal age.

At the time of interview, most participants had no embryos remaining in storage, though five reported having aneuploid embryos left and two of these expressed interests in further transfers. For 8 of 21 participants, the decision was explicitly described as a ‘last chance’ to achieve a biological child, typically after multiple failed cycles and limited embryo availability.

### Overarching theme: reclaiming possibility

Reflexive TA revealed an overarching theme of *Reclaiming Possibility*, describing how participants navigated uncertainty, institutional resistance, and moral responsibility in transferring embryos labelled ‘abnormal’ by PGT-A. Their accounts showed epistemic rupture and ethical reconstruction: moving from trust in clinical authority, through unlearning prior scientific guidance, to asserting personal judgement and reproductive intent. We constructed this theme through both explicit narratives and interpretive analysis, with four interconnected subthemes representing distinct expressions of reclaiming possibility.

**Figure 1.**
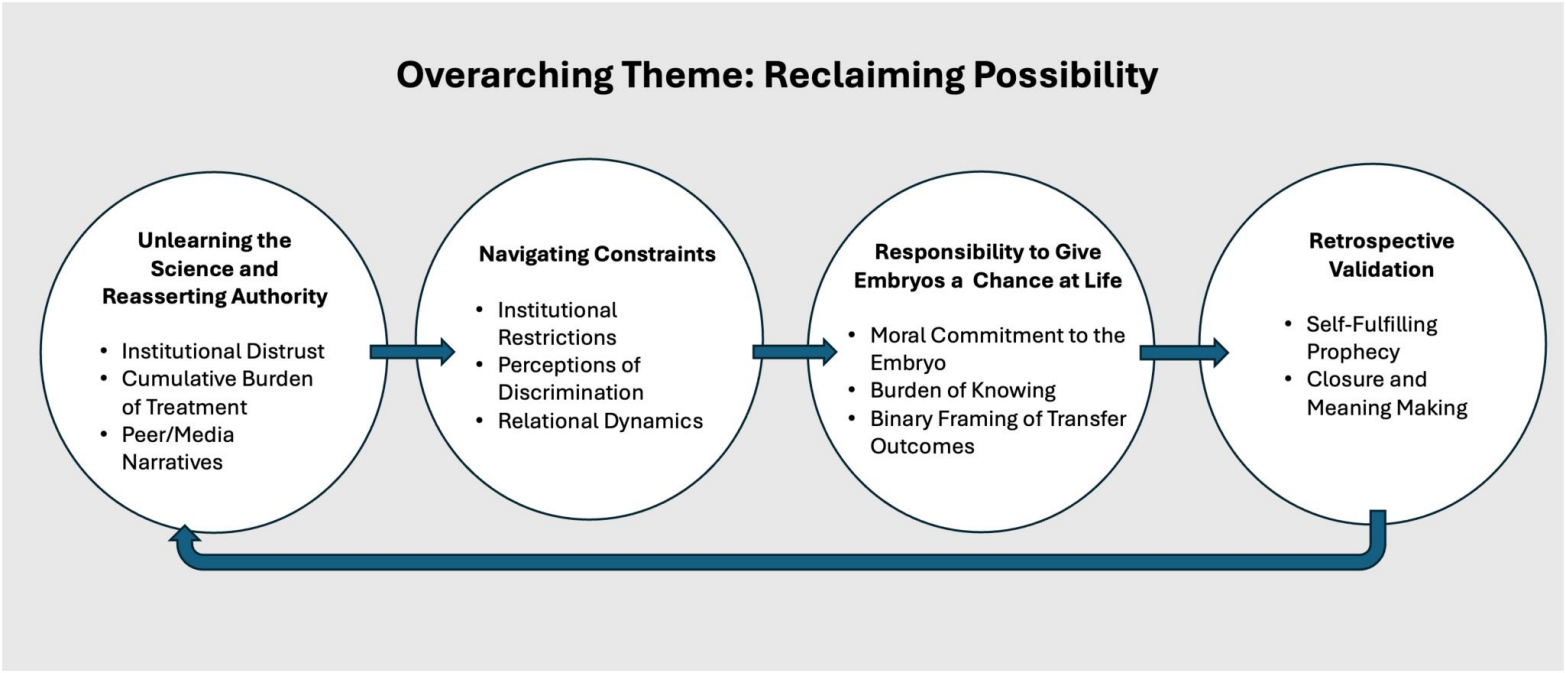
**Thematic process map of reclaiming possibility**. This figure presents a thematic process map showing how participants constructed and reinterpreted moral meaning across their fertility treatment journeys. *Reclaiming Possibility* refers to an evolving orientation through which participants sustained a sense of agency and moral coherence in the face of uncertainty and unfavourable outcomes. Four interrelated phases are depicted: (1) Unlearning the Science and Reasserting Authority; (2) Navigating Constraints; (3) Responsibility to Give Embryos a Chance at Life; and (4) Retrospective Validation. The directional arrows indicate movement between phases, while the curved arrow returning from Phase 4 to Phase 1 represents the recursive nature of the process, in which interpretations of past outcomes reshape future expectations and decisions.

As depicted in Fig. 1, *Reclaiming Possibility* functioned as a fluid, evolving orientation, sustained and reshaped through four interrelated phases: (i) reassessing scientific authority through unlearning; (ii) navigating institutional and relational constraints; (iii) embracing the responsibility to give—or withhold—embryos a chance at life; and (iv) retrospectively validating decisions through closure or moral justification. This orientation shaped and was shaped by each phase: questioning the test reopened parenthood; overcoming barriers enabled use of embryos; moral deliberation-centred responsibility—as one participant explained, ‘I’m not a religious person […]. But I don’t want to pick who gets to live and who gets to die’ (Case 5, 39 years, 2 IVF cycles, 2 live births)—and reflection reframed outcomes into a defensible ‘no-regrets’ stance. As another participant described: ‘I didn’t want to go through the rest of my life wondering, oh, could this have been, our daughter—and my daughter’s sister—if I didn’t give that a chance’ (Case 1, 38 years, 2 IVF cycles, SAB, implantation failure). Thus, *Reclaiming Possibility* functioned both as the unfolding process and as the desired outcome, even amid uncertain or unfavourable results.

### Phase 1: unlearning the science and reasserting authority

Participants described a gradual erosion of trust in PGT-A. This process of unlearning was shaped by three key factors: institutional distrust, the cumulative burden of treatment, and exposure to peer or media narratives of successful transfers involving embryos labelled ‘abnormal’.

#### Institutional distrust

Many participants perceived PGT-A as embedded within a routinized, industry-driven system. Accounts referenced policy variability across clinics (eligibility criteria, consent forms, and transfer policies) and limited pre-test counselling and unclear post-result pathways:

‘When I talked to the genetic counsellor, for me, it was just routine, checking a box. I came to not care what they said—because they’re paid by the company they work for, and that company wants you to have only one “normal” embryo. That way, they can discard the rest, and you’ll have to come back and pay for more cycles’ (Case 5, 39 years, 2 IVF cycles, 2 live births).

In addition, repeated IVF failures—often following the transfer of ‘normal’ embryos’ led participants to question the predictive value of PGT-A as well as the IVF protocol itself. This scepticism was not rooted in ignorance but in lived experience:

‘There’s a bias in the industry. Our Reproductive Endocrinologist (REI) once said: “Everything I’ve transferred has come out exactly like the PGT-A reported”, and I replied: “But you transferred a ‘normal’ boy embryo, and it turned into a blighted ovum. So clearly it wasn’t normal”’ (Case 4, 44 years, 2 IVF cycles, SAB).

All patients reported never consenting to a ban on transferring ‘abnormal’ embryos. In some clinics, physicians supported transfer but were constrained by institutional policy, forcing patients to navigate these restrictions elsewhere.

#### Cumulative burden of treatment

Participants described the psychological, physical, and financial toll of IVF. This burden was amplified by information from PGT-A, leaving many trapped in cycles of escalating interventions with diminishing returns.

Knowing the embryo’s genetic profile made failures feel deeply personal. Many resisted clinic framings of embryos as genetically worthy or unworthy, instead referring to them as ‘he’ or ‘she’ and attributing individuality and potential beyond ploidy. For some, this resistance was reinforced by painful losses from ‘normal’ embryos, which felt like a betrayal of the technology. The ‘normal’ label, meant to reassure, heightened the expectations, thus deepening disappointment when outcomes fell short.

‘After the first one, I wasn’t too upset—sometimes embryos just don’t implant. The second one was harder because we miscarried her. We were so hopeful because the test had said it was ‘normal’’ (Case 6, 46 years, 5 IVF cycles, chemical pregnancy).

One patient who terminated a pregnancy due to severe defects after a euploid transfer described being doubly burdened, by the loss itself and by the false reassurance of a ‘normal’ label.

‘And when my husband saw that IVF was just failing and failing, and I was devastated—especially after ending a pregnancy from a ‘normal’ embryo. Just so devastated to lose him. My husband said: ‘I can’t see you like this anymore. Even if we have an unhealthy baby, I just can’t see you like this anymore’’ (Case 14, 47 years, 4 IVF cycles, 1 live birth).

#### Peer/media narratives

While this interview study was performed, there had been several articles in the press, gaining media attention. Participants had done a search for the validity of PGT-A from a range of sources including doctors, peers, media coverage, medical journals and online and concluded that the test was unreliable.

‘And I have another doctor friend that transferred his wife one ‘aneuploid’, and they have a little girl, very healthy. So that’s the reason I believe that even though it is ‘aneuploid’, there’s always a chance to become a healthy baby’ (Case 2, 41 years, 6 IVF cycles, implantation failure).

These sources seemed to have initiated the question of the validity of PGT-A, inspiring participants to consider transferring ‘abnormal’ embryos, contributing to a sense of hope and increasing uncertainty about embryo disposal.

### Phase 2: navigating constraints

#### Institutional restrictions

Clinics often refused to transfer ‘abnormal’ embryos, citing liability or policy without explanation. When participants questioned PGT-A and sought transfer of ‘abnormal’ embryos, they encountered systemic resistance through institutional barriers and pushback from clinicians or partners. Many questioned why embryos were preserved if transfer was never intended:

‘They just told me: ‘They’re not good’, And I said: ‘Well, what about the rest of them?’. And they said: ‘Oh, they weren’t good either’. That’s all they said. And at that point, you want to believe what your doctor is telling you, you know? So, I said: ‘Okay’. But I kept wondering, if they weren’t good, why were they kept? Is it common for the clinic to store embryos that are considered not viable?’ (Case 5, 39 years, 2 IVF cycles, 2 live births).

Institutional refusals, liability concerns, and performance metrics shifted choices and costs onto patients, narrowing transfer pathways and externalizing the burdens of uncertainty:

‘And this is, frankly, optional medical care. So, it’s a little different than ‘Hey, Doctor, can you do your heart surgery a little bit differently’. We’re talking about an embryo that you’re going to discard anyway. So, the risk of damage, the risk–reward balance, didn’t make any sense to me’ (Case 5, 39 years, 2 IVF cycles, 2 live births).

The participants also described feeling ‘rejected’ or ‘dismissed’ by providers, particularly when exclusion was based on specific chromosomal labels rather than individualized assessment:

‘And then she (the director) let me know about a month later that they were not comfortable (with an embryo transfer), not because of policy, but because it was ‘Trisomy 14’, which, you know, potentially could result in some sort of disability with the child. So that was their rationale’ (Case 1, 38 years, 2 IVF cycles, SAB, implantation failure).

#### Perceptions of discrimination

Several participants described a sense of exclusion by fertility institutions due to the PGT-A results. One couple, who had sought treatment in multiple countries, expressed frustration that the act of testing itself, rather than an individualized appraisal of prognosis, led to transfer being declined due to the label:

‘We feel it is just discrimination. If we hadn’t tested, they would have happily transferred the embryos. Because we tested, they will not. We cannot transfer these embryos to another place because the law does not allow it. We will sign waivers, whatever, but they will not do it… so hard!’ (Case 20, 44 years, 30 IVF cycles, SAB).

To this participant, the refusal to transfer embryos solely based on a test result constituted a form of genetic discrimination.

#### Relational dynamics

Some participants reported initial disagreement with their partners, who feared miscarriage or disability. For some, it was about the psychological devastation that the patients were in to accept the risk.

‘Honestly, it was the toughest decision I’ve ever made. It wasn’t a simple yes-or-no binary, it was filled with fear and anxiety. I had to imagine a life very different from what I had pictured for myself and my family. The decision point became: do I accept the possibility of a (fourth) child with abnormalities and a happier wife, or keep saying no and just have my three healthy children and an unhappy wife?’ (Case 15, 47 years, 4 IVF cycles, 1 live birth).

Several participants reported that clinicians or family members tried to dissuade them, framing the choice as irrational or dangerous. Still, they persisted, citing a moral duty to the embryo or a personal commitment to try everything possible.

### Phase 3: responsibility to give embryos a chance at life

Decisions to proceed with the transfer of ‘abnormal’ embryos were presented as reasoned acts, which many framed in moral terms, asserting parental responsibility over clinical prognostication.

‘I was having a hard time morally. Those embryos were mine and my partner’s. We knew the gender, we knew the chromosomes—to us, those were our babies. I said: ‘If they were going to die, they were going to die with me as their mother’’ (Case 16, 34 years, 5 IVF cycles, implantation failure).

After repeated IVF failures, exhaustion, and institutional resistance, patients reaffirmed their goal of parenthood: as one put it, accepting the risks was simply ‘better than no baby at all’.

‘Giving a try is simply better than no baby at all… I have been through so much. So, I have nothing else to lose. I can’t be more hurt by the process’ (Case 19, 47 years, 5 IVF cycles, 6-day post-transfer).

While fear of disability and uncertain outcomes remained, these concerns were held in tension with the desire to give the embryo a chance and the fear of future regret. Many ultimately decided to proceed based on a pragmatic acceptance of uncertainty and the prospect of anticipated closure, rather than confidence in PGT-A’s determinacy.

‘I think we also knew that we would never regret it IF we tried. If we didn’t try, we would always wonder: what if?’ (Case 16, 34 years, 5 IVF cycles, implantation failure).

Three interrelated subthemes illustrate how participants came to view this transfer as a final, yet meaningful, act of reproductive agency: giving a chance to life, the burden of knowing and coping with uncertainty about the binary framing of embryo fate.

#### Moral commitment to the embryo

Participants described a dilemma: the burden of transferring an ‘abnormal’ embryo versus the anguish of discarding what felt like their only path to genetic parenthood. For many, disposal was not clinical routine but a profound personal loss.

‘I mean, we had had so many losses, I just didn’t feel right throwing it in the trash or even donating the embryo to research. We would prefer not casting her off as if it was just trash in the clinic because she didn’t have the right genetic makeup’ (Case 6, 46 years, 5 IVF cycles, chemical pregnancy).

‘It sounds somewhat morbid, but my husband and I basically decided: if she’s gonna die, we want to give her the best chance of life possible’ (Case 12, 44 years, 4 IVF cycles, 1 live birth).

Obligation, love, and grief led many to embrace uncertainty, not by denying risk, but by accepting it and allowing nature, rather than policy, to decide the outcome. For some, this choice intersected with gender preference: when PGT-A was used for sex selection, participants weighed transferring an ‘abnormal’ embryo of the desired sex against a ‘normal’ one of the opposite sex, ultimately reaffirming their intent.

‘We just wanted to try the girl (labelled as abnormal) first, period, because if she took, then that would have been what we had initially set out to accomplish’ (Case 11, 38 years, 1 IVF cycle, implantation failure).

In this way, participants returned to the motivations that had led them to seek fertility treatment in the first place: not to select for health, but to have a child. Even when chances of success were low, transfer was framed as better than doing nothing. A final act of hope.

#### Burden of knowing

Despite viewing the transfer as ethically justified, participants also described the heavy emotional burden of knowing the embryo’s genetic status. For some, this knowledge complicated—rather than clarified—their decision-making. Several expressed a wish that they had never undergone PGT-A, as they might have otherwise transferred the embryos without hesitation.

‘I almost wish that I maybe would have gone into this blindly and that one of those embryos would have resulted in a pregnancy, a life that would have been what was meant to be’ (Case 1, 38 years, 2 IVF cycles, SAB, implantation failure).

The burden was greatest for those whose natural pregnancies or ‘normal’ embryo transfers still ended in miscarriage or poor outcomes. For them, PGT-A did not remove uncertainty but added a new risk, demanding decisions without reassurance. With clinicians stressing low success rates, participants weighed medical probabilities alongside the financial, physical, and emotional costs to themselves and their families.

‘It’s scary to risk paying for a transfer that you’re less sure is going to work. And then, if it does take, perhaps you have a higher risk of early miscarriage. And if you don’t have an early miscarriage, perhaps there is something wrong with the chromosomes, and you won’t find out until you’re in your second trimester. So, it was definitely nerve-wracking. Triple times over: miscarriage, something wrong, termination’ (Case 10, 38 years, 1 IVF cycle, implantation failure).

Still, many emphasized the fear of future regret: if they chose not to try, they would always wonder what might have been. They justified it as the same as risk of a natural pregnancy. For them, the act of transfer became not just a clinical decision, but a reassertion of agency within a system that had repeatedly failed to deliver on its promises.

#### Binary framing of transfer outcomes

To manage uncertainty, many participants framed embryo fate in binary terms: either a healthy child or failure. This simplified perspective, often reinforced by thorough consent and empathetic counselling, helped them navigate the limits of predictive accuracy. Participants valued clinicians who explained evidence respectfully, contrasting with prior dismissive care. Reducing risk to two outcomes gave them clarity and psychological preparedness for whatever followed.

‘It was either going to be a healthy baby, or it wasn’t going to stick. I had to believe that. That belief made it bearable’ (Case 1, 38 years, 2 IVF cycles, SAB, implantation failure).

For some, this binary logic provided ethical relief, a way to shift the weight of responsibility from themselves to something larger: nature, fate, or the body itself.

‘I just kept thinking: if it’s not meant to be, it won’t be. But at least I’ll know I gave it a chance’ (Case 4, 44 years, 2 IVF cycles, SAB, implantation failure).

‘This sort of ethical concern about putting your wife through this, about creating a moment of pain, was all sort of dissolved by: well, it’s going to work, or it’s not going to work’ (Case 9, 45 years, 1 IVF cycles, implantation failure).

This framing let participants act without certainty; transfer became a symbolic and moral resolution in accepting the uncontrollable. One couple likened transferring a PGT-A-labelled ‘trisomy 21’ embryo to Schrödinger’s cat (Trimmer, 1980), its potential unknowable until tested: ‘You don’t know if it’s alive or dead unless you open it’ (Case 21, age 44, 30 IVF cycles, SAB). It was their first pregnancy after repeated failures with ‘normal’ embryos, though it ended in miscarriage at 6 weeks.

### Phase 4: retrospective validation

Retrospective validation was less an endpoint than an ongoing reappraisal, sometimes anticipatory, that fed back into earlier phases of *Reclaiming Possibility*. Regardless of outcome, participants integrated their choices into a coherent narrative of closure, without restoring confidence in PGT-A as a deterministic guide.

#### Self-fulfilling prophecy

Those who achieved live births following ‘abnormal’ transfer often described a sense of inner conviction or intuition, a belief, sometimes unexplainable, that the embryo would succeed despite its label:

‘I knew that it was going to be. I just don’t know how to explain it… I’m very data-driven. My feet are deep in the ground. I’m extremely realistic… but I knew it. I never doubted for a second it wasn’t going to work’ (Case 14, 47 years, 4 IVF cycles, 1 live birth).

‘Sometimes it’s intuition, it’s guidance, it’s… whatever it is. Something leads you there. Like meeting my wife. So, this is kind of how I felt with the doctor and the embryo and the journey that we ended up with’ (Case 12, 44 years, 4 IVF cycles, 1 live birth).

Even when transfers failed, participants described the act as meaningful, offering ‘peace’, ‘closure’, or ‘no regrets’. For some, it became a self-fulfilling prophecy, trusting the embryo and choosing to proceed gave the experience value regardless of outcome.

‘We felt a peace that, no matter what happened with those transfers, it was meant to be. It was kind of a Hail Mary, but choosing to go through with it made it feel right, like it had purpose, even if it didn’t work’ (Case 16, 34 years, 5 IVF cycles, implantation failure).

#### Closure and meaning making

Remarkably, failed implantation or miscarriage did not restore participants’ belief in PGT-A. Instead, they interpreted loss as chance or bodily factors, allowing them to move forward without regret. Even Case 19, interviewed 6-day post-transfer, described closure: she had ‘nothing else to lose’, framing transfer as a meaningful culmination. Importantly, participants did not view failed transfers as misguided but as providing emotional closure rather than validating the test.

‘It didn’t make me a half-believer in the test. I think the test can get it right. But just because the test gets it right once, doesn’t mean it gets it right reliably… You can close your eyes and throw a dart and hit a bullseye by accident. You know what I mean?’ (Case 10, 38 years, 1 IVF cycle, implantation failure).

Some found failure with ‘abnormal’ embryos more bearable than with ‘normal’ ones, as it felt less like personal fault, aligned with test predictions, and reflected a choice made with full awareness and agency as intended parents.

‘But working with the clinic, going through the science of ‘abnormal’ embryos, and all of this… It brought us together, too. There’s something… I don’t know, almost a perverse positive outcome to loss when you go through it with a partner’ (Case 4, 44 years, 2 IVF cycles, SAB, implantation failure).

Participants did not dismiss risk or science but redefined responsible reproduction. With clinical certainty failing them, they reclaimed decision-making based on values, where possibility was not conferred by medical authority but reclaimed through experience, loss, and belief.

Yet even successful outcomes carried a subtle burden. Although participants who had live healthy births felt profound gratitude, some described persistent low-level anxiety at developmental milestones—a vigilance absent with their other children. One mother whose daughter was thriving described the lingering shadow of the ‘abnormal’ label.

I think every time she reaches a milestone, like counting, talking, you know, running, walking. I think I will always, always be on the lookout, although there is nothing to be on the lookout for. She’s, thank God, a normal, beautiful, healthy, smart baby. (…) I’m like 99.9% it’s not relevant anymore, but there is always going to be a tiny fear in me that I didn’t have with my other children. A fear in me and in my husband too. I’m sure that there’s always going to be, Oh, my God, what if. What if? What if? What if? (Case 14, 47 years, 4 IVF cycles, 1 live birth).

To manage this ongoing uncertainty, these parents cyclically returned to Phase 1 reasoning—reassessing PGT-A’s limitations and their original ethical justification. This recursive engagement reinforced their conviction that transfer had been the right choice, demonstrating that ‘reclaiming possibility’ is an ongoing process extending beyond birth. The label, once acquired, cannot be unlearned—only continually reinterpreted.

Overall, these results present thematic patterns illustrated through participants’ own words, with broader interpretation and policy implications explored in the Discussion.

## Discussion

### Overview

This qualitative study sought to explore the motivations, decisional processes, and ethical reasoning of patients who transferred embryos labelled ‘abnormal’ by PGT-A at our centre after refusals elsewhere. It serves as a continuation of our historical learning process from the practice of transferring ‘abnormal’ embryos. In 2022, we reported eight live births following the transfer of 144 embryos labelled as abnormal by PGT-A (Barad *et al.*, 2022), seven of them healthy babies, and one born with a seemingly unrelated aortic coarctation that was surgically corrected uneventfully as a newborn. Since then, further healthy births from such transfers have shown that predicted chromosomal abnormalities often do not manifest, underscoring the test’s overstated predictive value, but no reports have ever been previously published studying patients’ perspectives on such transfers.

In contrast to our centre’s now over 10 years old practice, some clinics only in recent years started allowing selected mosaic transfers, while fully aneuploid embryos remain almost universally excluded from transfers. Earlier patient-centred studies on mosaic embryo transfers identified need for clearer information, detailed statistics, and peer support (Cheng *et al.*, 2022). Our patients who, most notably, at times transferred embryos by PGT-A reported as ‘fully’ aneuploid (which in reality—as by now is well understood—may only be mosaic embryos to variable degrees)—echoed these needs, and often sought information independently, which often could be contradictory, or relied on media reports of healthy births following transfer of abnormally-labelled embryos.

While sharing some decisional factors with mosaic transfers, these cases involved greater institutional resistance, often after multiple indisputable refusals ‘because of policy’, requiring sustained advocacy to challenge entrenched norms. They also presented greater relational constraints with partners or families, factors not discussed in prior studies. For those forced to ship embryos to other clinics, sometimes across borders, the drive to reevaluate treatment options was even stronger (Cases 20–21). For these patients, reclaiming possibility demanded both a binary framing, embryo as viable or not, and an extraordinary commitment.

Considering the coexistence of advanced maternal age (mean 38 years) and multiple failed IVF cycles (average of five), for most participants in our cohort, ‘abnormal’ embryos were not an ethical dilemma but their last chance for a child, making institutional policies in fertility clinics paradoxically a barrier to reproductive possibility itself. Logically, the remainder of this discussion addresses PGT-A’s clinical utility in relation to its scientific uncertainty, failures in informed consent, the ethics surrounding procreative beneficence, institutional and commercial pressures, and emerging legal and regulatory challenges.

### The clinical dilemma of managing scientific uncertainty

The core issue is not merely that PGT-A is imperfect, but that its residual uncertainty directly generates management dilemmas in clinical practice. When results reported ‘abnormal’ findings (including low-level mosaicism or segmental aneuploidies), professionals face incongruent directives, aiming to protect outcomes by avoiding transfers deemed risky and at the same time respecting patients' autonomy when these embryos may still be viable, all while maintaining consistency with institutional policies and liability frameworks.

In a recent evaluation of embryo selection strategies, including PGT-A, across 40+ years of IVF practice, one has to conclude that despite nearly 37 000 publications, embryo selection remains an unproven hypothesis, with cumulative pregnancy and live birth rates in unselected populations in principle not having improved and even the possibility of shortening time to pregnancy applying only to very good-prognosis patient cohorts who usually produce good numbers of eggs as well as embryos (Gleicher *et al.*, 2025b).

In September of 2024, the ASRM/SART Practice Committees, finally, formally and explicitly acknowledged that, so-far, PGT-A as a routine screening test has not been demonstrated to improve any IVF outcome parameters and noting multicentre randomized trials with overall outcomes similar to conventional IVF and an unclear effect on miscarriage risk (ASRM, 2024).

Yet. the ASRM/SART document declined to offer prescriptive guidance: it neither individualizes indications nor discourages routine use of PGT-A, thereby outsourcing decision-making to local policy and leaving clinicians and patients without harmonized standards (ASRM, 2024). In day-to-day practice, however, the interpretation of an ‘abnormal’ report is—despite its known limits—treated as determinative. The result is, of course, that patients encounter heterogeneity across clinics—as conflicting rules rather than transparent probabilities rule the day, which is precisely where scientific uncertainty becomes a medical care problem.

Once labelled, embryos usually move into an administrative category from ‘ethical disposal’ to simply ‘non-transferable’ or ‘low priority’, which, often, short-circuits shared decision-making. Patients who understand that PGT-A can misclassify developmentally competent embryos then confront a paradox: the test meant to optimize their outcomes in IVF—now prevents their very attempt at pregnancy through IVF. This is not simply an epistemic concern, it erodes confidence in counselling, restructures choices (discard vs store vs transfer elsewhere) and redistributes costs (additional cycles, shipping embryos out, etc.).

Our data show that several patients who were told they had ‘no transferable embryos’ ultimately achieved healthy pregnancies after transferring the same embryos at one centre with different policies. This dissonance forces a deeper question: if uncertain results systematically trigger prohibitions and further treatment cycles, does the initial indication for testing remain justified for these patients? In such a context, insisting on test-driven exclusion may invert beneficence, privileging risk minimization over the possibility of pregnancy, particularly for those with limited embryo numbers.

The practical challenge is not to rehearse technical limitations already known, but to align result reporting and policy with uncertainty-aware care. This would include expanded counselling on interpretive ranges, explicit pathways for ethically permissible transfer under consent, and institutional guardrails that avoid defaulting uncertain results into categorical bans. In short, uncertainty should trigger deliberation, not automatic exclusion.

### Patient perspectives and failures in informed consent

Interestingly, most participants in our study were initially not sceptical of PGT-A. Doubts, however, arose after lived experiences, such as failed implantations, miscarriages following euploid transfers, or being told there were ‘no transferable embryos’. The patients’ responses were not a rejection of science, but more a disillusionment with the routinized use of a technology lacking adequate validation and a reasonable request for clarity.

A common theme was information asymmetry. Patients were informed about potential benefits (higher pregnancy rates, fewer miscarriages), but less often about the test’s interpretive limits or how uncertain results would be managed. When outcomes disappointed, the information PGT-A provided, such as sex, chromosomal calls or implied ‘fitness’, intensified grief rather than prevented it. From the perspective of non-maleficence, this represents an ethical failure: risks were neither adequately studied nor conveyed. This suggests that informed consent should not only list risks and benefits but also prepare patients for uncertainty and outline credible pathways if results are equivocal.

Crucially, this counselling must occur upstream, before ordering PGT-A, so that patients can decide whether testing aligns with their goals and tolerances for uncertainty. Post-test counselling is necessary but insufficient to repair harm once options have already been constrained by a label. Accordingly, counselling might include the following: expected probability ranges (not just labels), contingency plans for ‘abnormal’ results (transfer options, storage, or further testing), and a candid discussion of financial and emotional costs over multiple cycles. Framed this way, consent becomes a pre-commitment to navigate uncertainty and a tool for shared planning rather than a formality, aligning with common-sense expectations patients bring to care.

Beyond the transfer decision, failures of informed consent have consequences extending into parenting. Our data suggest that labelling embryos as ‘abnormal’, reinforced by institutional refusal, can seed reflexive discrimination (Lippert-Rasmussen, 2013). Parents repeatedly re-engage the label at milestones (walking, talking, learning) producing heightened surveillance that differentiates the labelled child from siblings despite equivalent health. This label-driven vigilance is subtle but lingering and shapes parenting and persists long after birth, showing how clinical classification frameworks migrate into everyday care. Recognizing this dynamic justifies anticipatory counselling that names the risk, normalizes the impulse, and offers strategies to decouple milestone monitoring from label-primed anxiety. While larger studies should examine prevalence and duration, clinics must recognize that embryo labelling, and transfer refusals can precipitate long-term psychosocial harms.

#### Procreative beneficence

Procreative beneficence proposes selecting the child expected to have the best chance of a flourishing life (Savulescu, 2001). In practice, procreative beneficence is intended to guide prioritization when several embryos are available. Its relevance changes, however, when no euploid embryos exist or when test results are uncertain. In those contexts, the ethical question is not which embryo is best, but rather whether attempting pregnancy with available embryos is permissible under robust consent.

Our institutional stance reflects this distinction: we strongly advise against transferring embryos with aneuploidies known to be compatible with live birth of significantly affected offspring (e.g. trisomy 21, 18, 15, 13, certain sex chromosome anomalies), yet we may proceed after intensive counselling and an ‘against medical advice’ signed informed consent when patients, understanding risks, have no realistic alternatives and, nevertheless, decide to proceed against our best medical advice. Ethically, this aligns with the principle that ‘ought implies can’: Procreative beneficence cannot require a choice that does not exist (Britannica, 2025).

Crucially, procreative beneficence should not be used as a retrospective veto after testing has already constrained options. Given the predictive uncertainty of PGT-A, categorical prohibitions are hard to justify. In their place, therefore, should be graduated recommendations linked to phenotype severity, patient context, and clear prenatal diagnostic plans that better fit the principle’s original spirit.

#### Institutional and commercial pressures

Patients also often encountered refusals framed in terms of liability, centre policy, or performance metrics only after PGT-A was recommended and/or already performed. This sequencing matters: it shifts uncertainty and costs to patients (additional cycles, storage fees, cross-clinic shipping, etc.) while centres remain aligned with live-birth reporting marketing-cantered incentives.

Unsurprisingly, participants, therefore, also perceived conflicts of interest, noting that repeat cycles with PGT-A can be revenue-generating while centres often present themselves and are judged by their aggregate success rates.

At a systemic level, the refusal to transfer ‘abnormal’ embryos, despite the patient’s willingness and understanding of the risks, may be seen not only as a paternalistic application of ethical principles but also as one aligned with institutional and commercial interests (Patrizio *et al.*, 2025). The principle of procreative beneficence thus becomes entangled with commercial and reputational incentives, raising serious concerns about how ethical ideals are operationalized in practice.

#### Legal and regulatory considerations

Recent legal as well as professional developments highlight the urgency of recalibrating the role of PGT-A. In the USA, multiple class action lawsuits (2024–2025) accuse—at this stage in principle only selected major testing laboratories—of misleading marketing by claiming ca. 96–99% accuracy for the test, reduced miscarriage rates, and faster time to pregnancy, while downplaying limitations despite evidence that PGT-A cannot reliably distinguish viable from nonviable embryos, especially if mosaic or exhibiting segmental abnormalities (Ducharme, 2025; Freeman, 2025). At last reporting, nearly 700 patients had already participated.

These cases echo concerns that PGT-A’s rapid commercialization has outpaced both clinical validation and ethical oversight (Takahashi *et al.*, 2019). At the time of this report, several defendants have sought early dismissal, and all the filed lawsuits remain pending. They commonly assert consumer-protection and warranty claims and were filed across multiple jurisdictions. Media reports, moreover, suggested that additional defendants may be added and that filings may be expanded.

This litigation highlights a broad oversight gap: Despite its widespread clinical use in the USA and—as here outlined—its obvious very profound consequences on medical practice in the IVF field—the Food and Drug Administration (FDA) has completely ignored PGT-A. One reason may have been that, like other so-called ‘add-ons’ to IVF (Glatthorn and Decherney, 2022) PGT-A was brought to market as so-called laboratory-developed tests (LDT), a test category the FDA has historically abstained from reviewing. This, however, changed in May of 2024, when the FDA announced new rules for regulating LDTs (Medical Devices; Laboratory Developed Tests 2024), which were widely welcomed. Unfortunately, a Federal Court in March of 2025, vacated this new FDA rule, siding with the American Clinical Laboratory Association which had sued the FDA over this new policy (American Clinical Laboratory Association, 2025).

Laboratory regulations regarding IVF practice, therefore, remain a patchwork, leaving substantial latitude in how tests are marketed, and results are operationalized. And, unsurprisingly, patients described limited disclosure of uncertainty and institutional bans that restricted options. Although our legal discussion focuses on the USA, similar challenges have been noted internationally: for example, professional guidance in Europe (e.g. ESHRE) emphasizes standardized reporting and counselling for mosaic results (ESHRE Working Group on Chromosomal Mosaicism *et al.*, 2022), while national regulators (e.g. the UK HFEA) acknowledge clinic-level variability (HFEA, n.d.); a recent Canadian survey (Chan *et al.*, 2022) likewise reports marked heterogeneity with very limited transfer of embryos reported as fully aneuploid.

Respecting patient autonomy requires that this epistemic concern of knowing more but understanding less should be addressed through independent regulation, standardized and transparent reporting, consent processes that explicitly anticipate uncertain results and their management, and a reframing of ‘embryo viability’ within both clinic policy and professional guidance. Altogether, these steps would align practice with evidence, reduce centre-to-centre variability, and refocus policy on informed choice rather than implied certainty.

### Limitations

This study has several limitations. First, it is a single-centre qualitative study with a self-selected sample, which constrains transferability. Second, interviews were retrospective—sometimes years after transfer—so recall bias is possible; we attempted mitigation via timeline-anchored prompts and, when available, cross-checks with records. Third, duration of treatment and the number of prior IVF cycles may have influenced decision-making: participants with longer, unsuccessful histories often described current embryos as a ‘last chance’, potentially lowering their threshold for accepting uncertainty. Although this is a *post hoc*, qualitative observation rather than a tested association, it represents a potential source of decisional and selection bias in our sample. Fourth, participants could choose joint or separate interviews; although we compared theme expression across formats and did not observe format-specific themes, response bias cannot be excluded. Fifth, religious affiliation was not systematically collected. Although we noted when participants voluntarily mentioned religious identity (two religious, eight explicitly non-religious), this inconsistent data collection limits systematic analysis of faith-based determinants. Sixth, the legal and regulatory analysis is US-centred, though we briefly note international variability and have framed practice recommendations to be jurisdiction-agnostic. Finally, as a qualitative study, findings constitute analytic generalizations rather than statistical estimates; future work should include prospective registries capturing maternal, neonatal, and psychosocial outcomes after transfers involving ‘abnormal’ labels.

## Conclusion

When evidence is uncertain, reproductive care should default to deliberation rather than prohibition. The present study shows that categorical refusal to transfer embryos labelled as ‘abnormal’ by PGT-A can unnecessarily constrain reasonable pathways to parenthood. Especially when alternatives are limited, we propose uncertainty-explicit counselling, defined routes for transfer under robust consent, and clear prenatal diagnostic plans adjusted to a patient’s specific case.

Guidance should standardize reporting and reduce incentives for distortions. Future work should, moreover, prioritize prospective registries that track maternal, neonatal, and psychosocial outcomes after such transfers, and evaluate consent models that prepare patients for uncertainty. Ultimately, success should not only include outcomes, but whether patients’ choices are informed, coherent, and genuinely theirs.

## Supplementary Material

hoag025_Supplementary_Data

## Data Availability

The data underlying this article will be shared on reasonable request to the corresponding author.
