# Supplementary material for: Reclaiming ‘abnormal’ embryos after preimplantation genetic testing for aneuploidy: patients’ perspectives on transferring embryos against prior institutional advice
Source: Hum Reprod Open. 2026 Mar 26;2026(2):hoag025. doi: 10.1093/hropen/hoag025 (PMC13070650; doi:10.1093/hropen/hoag025)
Supplement: hoag025_Supplementary_Data [file hoag025_supplementary_data.docx]

**Supplementary File S1.** Initial set of interview questions

| - Please tell me about your experience with fertility treatment leading up to your decision to transfer “abnormal” embryos? - How did you feel about the transfer? - How do you feel about the outcome of your transfer? - Were you aware that “abnormal” embryos were available to you? - How do you feel about undergoing PGT-A? - How do you feel about recommending PGT-A to other infertile couples? - Did you think of the possibility of other alternative choices? - How does your partner feel about the embryo? (if applicable) - Who else influenced your choice? - Did you consult anyone to make this decision? |
| --- |
